# Effects of Continuous Bt Maize Cultivation on Soil Nutrient Content and Microbial Communities

**DOI:** 10.3390/plants15010112

**Published:** 2025-12-31

**Authors:** Xiaomin Liang, Donghua Zhong, Shuai Yan, Yuanjiao Feng

**Affiliations:** 1 Key Laboratory of Agro-Environment in the Tropics, Ministry of Agriculture and Rural Affairs, South China Agricultural University, Guangzhou 510642, China; 2 College of Natural Resources and Environment, South China Agricultural University, Guangzhou 510642, China

**Keywords:** Bt maize, continuous cultivation, bulk soil, rhizosphere soil, soil nutrients, microbial community composition

## Abstract

The global population growth has driven the widespread adoption of genetically modified crops, with Bt maize, due to its insect resistance, becoming the second most widely planted GM crop. However, studies on the effects of continuous Bt maize cultivation on soil ecosystems are limited, and there is an urgent need to assess its ecological safety at the regional scale. To evaluate the potential effects of continuous cultivation of transgenic Bt maize on the soil ecosystem, a five-season continuous planting experiment was conducted using two Bt maize varieties (5422Bt1 and 5422CBCL) and their near-isogenic conventional maize (5422). After five consecutive planting seasons, bulk soil and rhizosphere soil were collected. The main nutrient contents of the bulk soil were measured, and high-throughput sequencing was employed to analyze microbial diversity and community composition in both soil types. The results showed that, compared with the near-isogenic conventional maize 5422, continuous planting of Bt maize varieties 5422Bt1 and 5422CBCL did not affect the contents of organic matter, total nitrogen, total phosphorus, total potassium, alkaline hydrolyzable nitrogen, available phosphorus, or available potassium in bulk soil. Regarding the microbial communities in bulk soil, there were no significant differences in the α-diversity indices of bacteria and fungi after five consecutive seasons of Bt maize cultivation, compared with soils planted with the near-isogenic conventional maize 5422. Proteobacteria and Ascomycota were the dominant phyla of bacteria and fungi, respectively. Principal coordinate analysis (PCoA) and redundancy analysis (RDA) revealed that the structure of microbial communities in bulk soil was primarily influenced by factors such as OM, TP, TN and AN, whereas the Bt maize varieties had no significant effect on the overall community structure. Regarding the rhizosphere soil microbial communities, compared with the near-isogenic conventional maize 5422, the evenness of the bacterial community in the rhizosphere soil of Bt maize decreased, leading to a reduction in overall diversity, whereas species richness showed no significant change. This change in diversity patterns further contributed to the restructuring of the rhizosphere soil microbial community. In contrast, the fungal community showed no significant differences among treatments, and its community structure remained relatively stable. Proteobacteria and Ascomycota were the dominant phyla of bacteria and fungi, respectively. Principal coordinate analysis (PCoA) indicated that continuous cultivation of Bt maize for five seasons had no significant effect on the structure of either bacterial or fungal communities in the rhizosphere soil. In summary, continuous cultivation of Bt maize did not lead to significant changes in soil nutrient contents or microbial community structures, providing a data foundation and theoretical basis for the scientific evaluation of the environmental safety of transgenic maize in agricultural ecosystems.

## 1. Introduction

With the continuous growth of the global population and the increasing prominence of food security issues, genetically modified (GM) crops have been widely adopted in agricultural production worldwide due to their superior stress resistance and high yield potential. By 2024, approximately 32 countries or regions had approved the cultivation of GM crops, with a total planting area reaching about 206 million hectares [1]. With the global expansion of genetic modification technology, Bt maize has rapidly increased in planting scale due to its highly effective insect resistance and has now become the second most widely cultivated genetically modified crop in the world, following only GM soybean [2]. Bt maize expresses toxin-encoding genes (such as cry1Ab and cry1Ac) derived from *Bacillus thuringiensis*, which effectively control a range of lepidopteran pests, including *Ostrinia nubilalis* and *Spodoptera frugiperda*. This confers enhanced resistance to insect pests, reduces the reliance on chemical pesticides, and provides substantial economic and ecological benefits in agricultural production [3,4,5].

Although Bt maize has achieved remarkable success in agricultural production, the ecological consequences of its continuous cultivation—particularly the potential impacts on soil ecosystems—remain a subject of considerable attention and debate worldwide [6]. In contrast to short-term studies, the continuous cultivation of transgenic crops under field conditions generates a cumulative ecological disturbance background, and its effects on soil microecosystems may be temporally delayed and subtle [7]. Under large-scale promotion and continuous cultivation, the toxin proteins secreted by Bt maize during its growth can remain in the soil through various pathways, such as pollen fall, root exudates, and plant residues. These residues may directly or indirectly affect soil nutrients, physicochemical properties, enzyme activities, soil fauna, and microorganisms [8]. Bt crops pose no significant risk to the environment or human health; most of the Cry proteins deposited in the soil can degrade within a few days, and newly planted crops grown in soil containing Bt Cry proteins do not absorb these proteins [9]. As the fundamental environment for crop growth, soil health is closely related to the structure and functional diversity of soil microbial communities, as well as to soil nutrient contents [10]. Soil microorganisms are a vital component of soil ecosystems, playing indispensable roles in nutrient cycling, organic matter decomposition, humus formation, and pollutant degradation [11]. Complex and reciprocal interactions exist between plant roots and soil microorganisms, whereby root exudates shape microbial community structure and function, while microbial activities, in turn, influence plant nutrient uptake and stress resistance [2,12]. Therefore, understanding whether continuous cultivation of Bt maize disrupts soil microbial diversity and ecological functions is crucial for evaluating the ecological safety of transgenic crops.

Currently, environmental risk assessments of genetically modified (GM) crops have primarily focused on non-target organisms and their diversity, gene flow, and the evolution of resistance in target pests, whereas studies on the effects of GM crops on soil ecological environments remain scarce [13]. Several studies have suggested that continuous cultivation of Bt maize does not significantly alter soil microbial community structure or diversity, nor does it cause noticeable changes in soil nutrient contents. A two-year field study demonstrated that, compared with its non-transgenic counterpart, the insect-resistant transgenic maize CM8101 showed no significant differences in rhizosphere soil properties, including moisture content, pH, urease activity, and acid phosphatase activity. This suggests that CM8101 had no observable adverse effects on the soil ecosystem, indicating a favorable environmental safety profile [14]. The results from a two-year study showed that, across three growth stages, there were no significant differences between the insect-resistant transgenic maize CM8101 and the non-transgenic maize Zheng58 in soil moisture content, pH, urease, phosphatase, and catalase activities, as well as in the structure and diversity of rhizosphere soil bacterial communities. These findings suggest that long-term cultivation of CM8101 does not significantly affect the major physicochemical properties, functional enzyme activities, or microbial community structure of the soil [15]. Similarly, Hong reported that the cultivation of insect-resistant and herbicide-tolerant transgenic maize had no significant effects on soil physicochemical properties or on the diversity and community composition of rhizosphere soil fungi [16]. Zeng reported that cultivation of transgenic salt-tolerant maize harboring the BcWRKY1 gene did not significantly alter key soil nutrient parameters, including AN, AP, and AK [17]. In a related study, Shuke demonstrated that the transgenic maize line 2A-7 exerted no significant influence on soil physicochemical characteristics, enzymatic activities, or microbial diversity, whereas the maize developmental stage was the predominant factor shaping these rhizosphere soil attributes [18]. At the same developmental stage, soil bacterial diversity did not differ significantly between the transgenic maize Ruifeng 125 and its non-transgenic counterpart. Further analysis indicated that the developmental stage, rather than the transgenic event, was the dominant factor driving changes in soil microbial diversity [4]. The observed inconsistencies among previous studies are likely due to inherent differences in maize genotypes rather than the influence of the introduced transgenes [19].

In this study, the ecological effects of continuous cultivation of Bt maize over five growing seasons were investigated. Bulk soil and rhizosphere soil samples were collected to determine the contents of key nutrients in bulk soil, and high-throughput sequencing technology was employed to analyze the diversity and structural characteristics of microbial communities in these two types of soils. These analyses provided a systematic evaluation of the effects of continuous Bt maize cultivation on the soil ecosystem. The objectives of this study were to: (1) assess the effects of continuous Bt maize cultivation on key nutrient contents in bulk soil and its potential influence on soil nutrient availability; (2) elucidate the effects of continuous Bt maize cultivation on the diversity and community structure of bacteria and fungi in both the bulk soil and the rhizosphere soil; and (3) examine the relationships between microbial communities and soil nutrient factors to explore the potential mechanisms by which Bt maize regulates soil ecological functions. The results aim to provide theoretical and practical insights for the scientific management of Bt maize cultivation, improved soil nutrient utilization, and the sustainable development of agricultural ecosystems.

## 2. Results

### 2.1. Nutrient Contents in Bulk Soil

As shown in Table 1, there were no significant differences in soil OM contents between the two Bt maize varieties (5422Bt1 and 5422CBCL) and their near-isogenic conventional maize (5422). However, a significant difference was observed between the two Bt maize varieties, with the OM content in the 5422CBCL treatment being 17.08% higher than that in 5422Bt1. In addition, no significant differences were detected in other major nutrient indices—including TN, TP, TK, AN, AP, and AK—between the Bt maize varieties and the conventional maize. These results indicate that continuous cultivation of Bt maize for five consecutive seasons did not significantly affect the soil nutrient status.

### 2.2. Diversity Analysis of Bulk Soil Microorganisms

#### 2.2.1. Unique and Shared OTUs in the Bulk Soil

**Figure 1:**
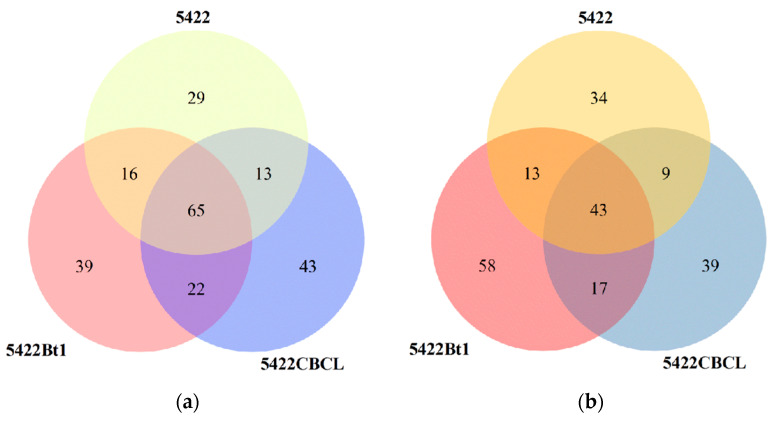
Shared and unique OTU numbers of bacterial (**a**) and fungal (**b**) communities in the bulk soil under different maize variety treatments.

Venn diagram analysis (Figure 1a) showed that a total of 65 bacterial operational taxonomic units (OTUs) were shared among the bulk soil of the three maize varieties. The Bt maize varieties 5422Bt1 and 5422CBCL had more unique bacterial OTUs (39 and 43, respectively) than the near-isogenic conventional maize 5422 (29). In the fungal community (Figure 1b), 43 OTUs were shared among the three maize varieties. The numbers of unique fungal OTUs in 5422Bt1 and 5422CBCL (58 and 39, respectively) were also higher than that in the near-isogenic conventional maize 5422 (34), with 5422Bt1 exhibiting the highest number of unique OTUs. Overall, both Bt maize varieties possessed a greater number of unique bacterial and fungal OTUs in the bulk soil compared with the near-isogenic conventional maize, and the two Bt lines differed in the abundance of their unique OTUs.

#### 2.2.2. α-Diversity of Microbial Communities in Bulk Soil

**Figure 2:**
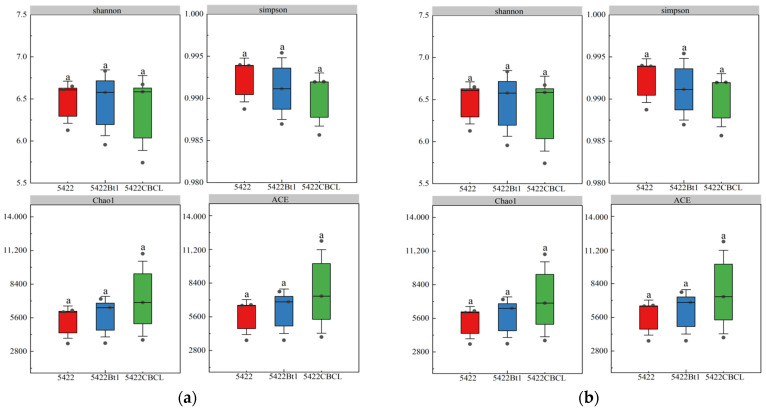
Effects of different maize variety treatments on the α-diversity of bacterial (**a**) and fungal (**b**) communities in the bulk soil. Different letters above the bars indicate significant differences among treatments (*p* < 0.05), whereas the same letters indicate no significant differences (*p* > 0.05).

Under continuous cultivation, no significant differences (*p* > 0.05) were observed in the Shannon, Simpson, Chao1, and ACE indices of the bacterial communities in the bulk soil among the three maize varieties (Figure 2a). Similarly, for fungal communities (Figure 2b), the Shannon, Simpson, Chao1, and ACE indices showed no significant differences (*p* > 0.05) among the different maize treatments, indicating that the overall diversity and richness of the fungal communities remained consistent under continuous planting. Overall, continuous cultivation of Bt maize did not significantly affect the α-diversity of bacterial or fungal communities in the bulk soil, suggesting that the introduction of the Bt gene and varietal differences did not notably alter the diversity structure of root-associated microbial communities. The microbial communities in the bulk soil exhibited strong stability under continuous cultivation.

#### 2.2.3. Composition of Microbial Communities in Bulk Soil

**Figure 3:**
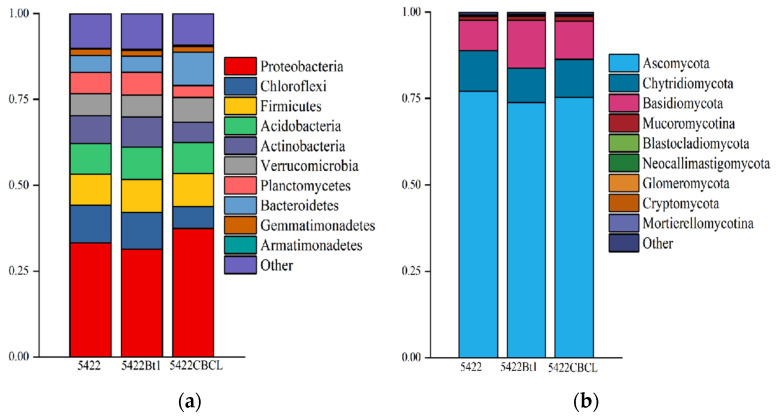
Phylum-level composition analysis of bacterial (**a**) and fungal (**b**) communities in the bulk soil under different maize variety treatments.

As shown in Figure 3a, Proteobacteria was the dominant bacterial phylum in the bulk soil of all three maize varieties, exhibiting the highest relative abundance, followed by Chloroflexi, Firmicutes, Acidobacteria, and Actinobacteria. The relative abundances of these major bacterial phyla showed no significant differences among the near-isogenic conventional maize 5422 and the two Bt maize varieties, 5422Bt1 and 5422CBCL, indicating that the bacterial community structure remained relatively consistent among the different maize varieties. The results of fungal community composition (Figure 3b) showed that Ascomycota was the overwhelmingly dominant fungal phylum in the bulk soil of all three maize varieties, accounting for more than 75% of the total fungal community. Chytridiomycota and Basidiomycota were the next most abundant phyla. No significant differences were observed in the relative abundances of the major fungal phyla among the three maize treatments, indicating that the overall fungal community composition remained consistent. Taken together, these results suggest that after five consecutive planting seasons, the dominant bacterial and fungal phyla in the bulk soil of Bt maize and the near-isogenic conventional maize were largely consistent, with no significant differences detected at the phylum level.

#### 2.2.4. β-Diversity Analysis of Microbial Communities in Bulk Soil

**Figure 4:**
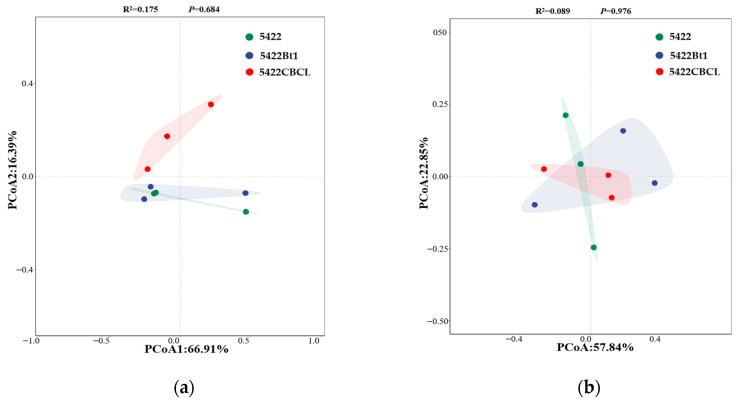
Principal component analysis (PCoA) of structural differences in bacterial (**a**) and fungal (**b**) communities in the bulk soil under different maize variety treatments.

For the bacterial community (Figure 4a), PCoA1 and PCoA2 explained 66.91% and 16.39% of the total community variation, respectively. Although a certain degree of separation was observed among the bacterial community samples from the bulk soil of the near-isogenic conventional maize (5422) and Bt maize lines (5422Bt1 and 5422CBCL) in the coordinate space, however, PERMANOVA analysis indicated that these differences were not statistically significant (R^2^ = 0.175, *p* = 0.684), suggesting that, overall, maize variety did not significantly alter the β-diversity of bacterial communities in root-surrounding soils. For the fungal community (Figure 4b), PCoA1 and PCoA2 explained 57.84% and 22.85% of the community variation, respectively. No clear separation was observed among the fungal community samples from the near-isogenic conventional maize 5422 and Bt maize lines (5422Bt1 and 5422CBCL) in the coordinate space, and the PERMANOVA results indicated that the differences among groups were not significant (R^2^ = 0.089, *p* = 0.976). Taken together, after five consecutive planting seasons, no significant differences were observed in the β-diversity of bacterial and fungal communities in the bulk soil between the Bt maize lines and their near-isogenic conventional maize. These results suggest that continuous cultivation of Bt maize exerts little influence on the overall structure of the root-associated microbial community.

#### 2.2.5. Effects of Environmental Factors on Bulk Soil Microbial Communities

**Figure 5:**
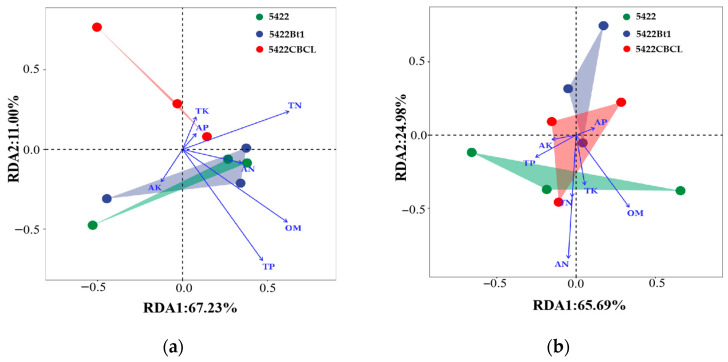
RDA of bulk soil bacterial (**a**) and fungal (**b**) communities and environmental factors.

In the RDA analysis of bacterial communities and environmental factors in bulk soil (Figure 5a), RDA1 and RDA2 explained 67.23% and 11.00% of the community variation, respectively. The results indicated that the bacterial communities under different maize varieties were influenced to varying degrees by environmental factors, among which TP, OM, and TN were the primary factors shaping the bacterial community structure. For the fungal community (Figure 5b), RDA1 and RDA2 explained 65.69% and 24.98% of the variation, respectively. The correlation analysis between environmental factors and the fungal community revealed that AN and OM were the key environmental factors driving the changes in fungal community structure.

#### 2.2.6. Correlation Heatmap Between Bulk Soil Microbial Communities and Environmental Factors

**Figure 6:**
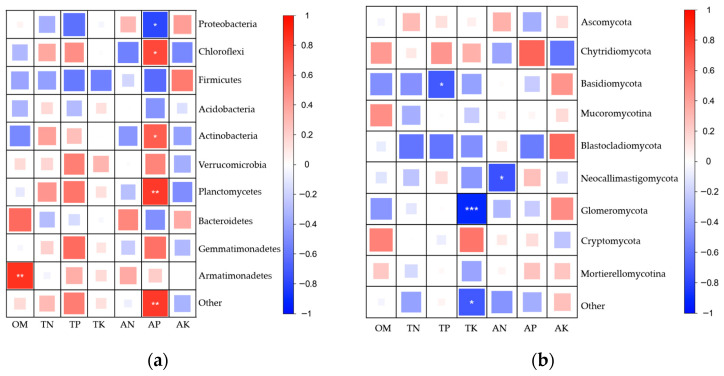
Heatmap showing the correlations between dominant microbial phyla and soil physicochemical properties under different maize treatments. Panel (**a**) represents bacterial communities, and panel (**b**) represents fungal communities. Color indicates the direction of the correlation, with red representing positive correlations and blue representing negative correlations. Both color intensity and square size reflect the strength of the correlation: darker colors and larger squares indicate stronger correlations, whereas lighter colors and smaller squares indicate weaker correlations. Asterisks denote statistical significance (* *p* < 0.05, ** *p* < 0.01, *** *p* < 0.001).

In the bacterial community (Figure 6a), Proteobacteria showed positive correlations with AN and AK, but negative correlations with TP and AP, suggesting that a high-phosphorus environment may inhibit the relative abundance of this phylum. Chloroflexi exhibited positive correlations with TP and AP, indicating that elevated phosphorus levels may enhance its abundance. In addition, Actinobacteria and Planctomycetes were significantly positively correlated with AP, while Bacteroidetes and Armatimonadetes showed significant positive correlations with OM, implying that higher AP or OM content may promote the enrichment of these bacterial groups. Conversely, Firmicutes displayed negative correlations with several nutrient indicators, suggesting that this phylum may be more sensitive to changes in soil nutrient conditions.

In the fungal community (Figure 6b), Glomeromycota showed a highly significant negative correlation with TK (blue, *p* < 0.001), representing the strongest correlation among all fungal phyla, indicating that TK content exerts a strong regulatory effect on the distribution of this group. Basidiomycota was significantly negatively correlated with TP (*p* < 0.05), while Neocallimastigomycota showed a significant negative correlation with available nitrogen (AN) (*p* < 0.05), suggesting that the relative abundance of these taxa decreases with increasing soil nutrient levels. In contrast, Ascomycota exhibited generally weak correlations with all measured soil physicochemical properties.

### 2.3. Rhizosphere Soil Microbial Diversity Analysis

#### 2.3.1. Unique and Shared OTUs in the Rhizosphere Soil

**Figure 7:**
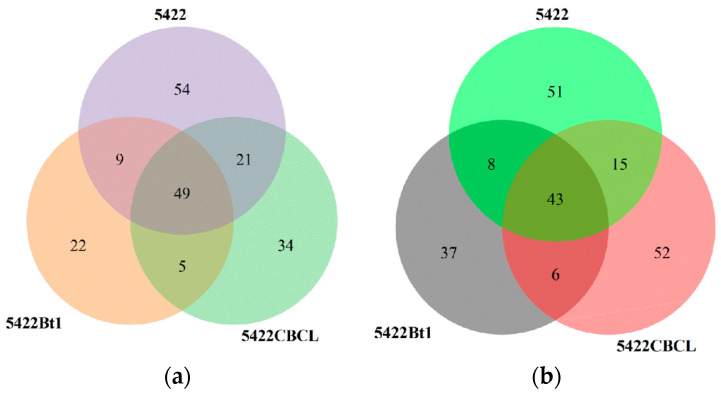
Shared and unique OTU numbers of bacterial (**a**) and fungal (**b**) communities in the rhizosphere soil under different maize variety treatments.

In the bacterial community (Figure 7a), a total of 49 operational taxonomic units (OTUs) were shared among the rhizosphere soil of the three maize varieties. The near-isogenic conventional maize 5422 possessed 54 unique bacterial OTUs, which was higher than those observed in the Bt maize varieties 5422CBCL (34 OTUs) and 5422Bt1 (22 OTUs). The rhizosphere soil bacterial community of conventional maize contained a greater number of unique OTUs than the Bt maize lines (5422Bt1 and 5422CBCL), and the number of unique OTUs varied among the Bt varieties. In the fungal community (Figure 7b), 43 fungal OTUs were shared among the three maize varieties. The near-isogenic conventional maize 5422 had 51 unique fungal OTUs, while the Bt maize varieties 5422CBCL and 5422Bt1 possessed 52 and 37 unique fungal OTUs, respectively.

#### 2.3.2. α-Diversity of Rhizosphere Soil Microbial Communities

**Figure 8:**
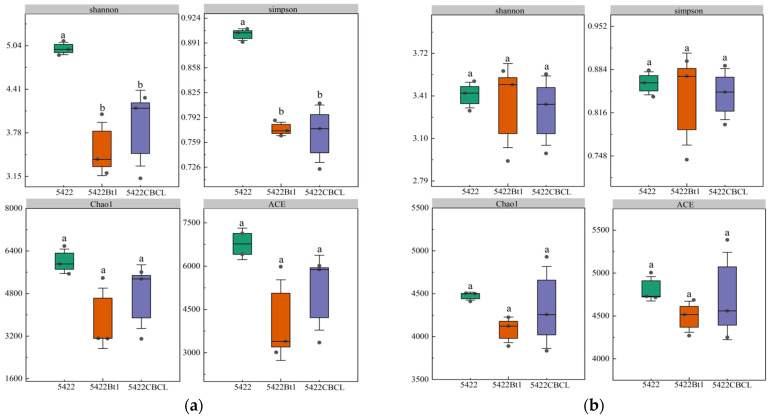
Effects of different maize variety treatments on the α-diversity of bacterial (**a**) and fungal (**b**) communities in the rhizosphere soil. Different letters above the bars indicate significant differences among treatments (*p* < 0.05), whereas the same letters indicate no significant differences (*p* > 0.05).

In the bacterial community (Figure 8a), Shannon and Simpson indices in the rhizosphere soil of the near-isogenic conventional maize 5422 were significantly higher than those of the Bt maize treatments (*p* < 0.05), whereas Chao1 and ACE indices did not differ significantly (*p* > 0.05). These results suggest that continuous cultivation of Bt maize slightly reduced community diversity in terms of evenness and diversity indices (Shannon and Simpson), but had little effect on species richness, as reflected by Chao1 and ACE.

#### 2.3.3. Composition of Rhizosphere Soil Microbial Communities

**Figure 9:**
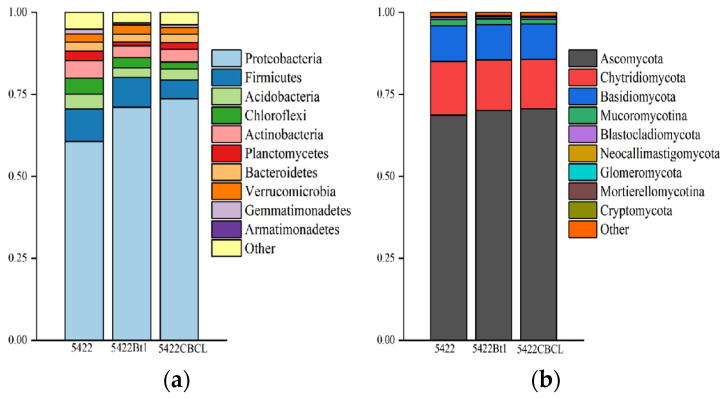
Phylum-level composition analysis of bacterial (**a**) and fungal (**b**) communities in the rhizosphere soil under different maize variety treatments.

At the bacterial community level (Figure 9a), the dominant phylum in the rhizosphere soil of all three maize varieties was Proteobacteria, which accounted for the highest relative abundance across all treatments. The relative abundance of Proteobacteria was significantly higher in the rhizosphere soil of Bt maize varieties 5422Bt1 and 5422CBCL than in the near-isogenic conventional maize 5422 (*p* < 0.05). Firmicutes and Actinobacteria were identified as the subdominant bacterial phyla. At the fungal community level (Figure 9b), Ascomycota was the predominant phylum across all treatments, occupying an absolutely dominant position within the fungal communities, followed by Chytridiomycota and Basidiomycota. The relative abundances of the major fungal phyla showed no significant differences among the maize varieties, indicating a stable overall community composition pattern. Overall, after five consecutive growing seasons, no significant differences (*p* > 0.05) were observed in the phylum-level composition of bacterial and fungal communities in the rhizosphere soil between Bt maize and its near-isogenic conventional counterpart. These results indicate that Bt maize had no substantial impact on the overall rhizosphere soil microbial community structure at the phylum level.

#### 2.3.4. β-Diversity Analysis of Rhizosphere Soil Microbial Communities

**Figure 10:**
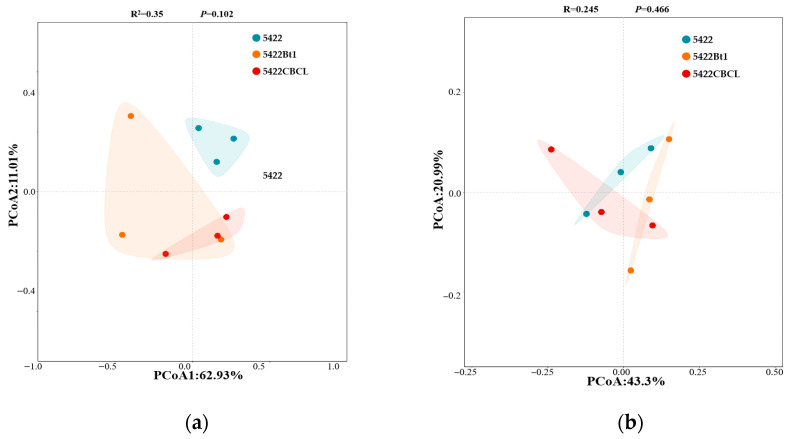
Principal component analysis (PCoA) of structural differences in bacterial (**a**) and fungal (**b**) communities in the rhizosphere soil under different maize variety treatments.

In the bacterial community (Figure 10a), PCoA1 and PCoA2 explained 62.93% and 11.01% of the total community variation, respectively. The bacterial community samples from different maize varieties exhibited a certain degree of separation in coordinate space, with Bt maize treatments distributed relatively independently from the near-isogenic conventional maize 5422 group. However, PERMANOVA analysis revealed that the differences among groups were not significant (R^2^ = 0.35, *p* = 0.102), indicating that the overall structure of the rhizosphere soil bacterial community did not differ markedly between Bt and conventional maize varieties. For the fungal community (Figure 10b), PCoA1 and PCoA2 explained 43.30% and 20.99% of the total variation, respectively. No clear separation was observed among fungal community samples of the different maize varieties, and the PERMANOVA results similarly indicated no significant differences among groups (R^2^ = 0.245, *p* = 0.466). Overall, after five consecutive growing seasons, no significant differences were observed in the β-diversity of bacterial and fungal communities in the rhizosphere soil between Bt maize and its near-isogenic conventional maize 5422. These results suggest that the maize variety had only a minor influence on the overall structure of rhizosphere soil microbial communities.

## 3. Discussion

With the widespread cultivation of genetically modified (GM) crops worldwide, their potential environmental safety risks have increasingly attracted attention. As the basis of plant growth, soil properties and changes in its physicochemical characteristics and microbial community structure are important indicators for evaluating the ecological risks of GM crops. In this study, near-isogenic conventional maize (5422) and two Bt maize varieties (5422Bt1 and 5422CBCL) were continuously cultivated for five seasons. We systematically analyzed their effects on soil nutrient status in the bulk soil, as well as the microbial diversity and community structure in both bulk soil and rhizosphere soil, in order to assess the ecological safety of Bt maize under continuous cultivation.

In terms of soil nutrients, after five consecutive growing seasons, the two Bt maize varieties (5422Bt1 and 5422CBCL) did not exhibit significant effects on the major nutrient parameters of the bulk soil compared with the near-isogenic conventional maize (5422), indicating that Bt maize did not markedly alter soil nutrient status under the current cultivation conditions. Notably, the soil OM content in the 5422CBCL treatment was significantly higher than that in 5422Bt1 and showed an increasing trend relative to the conventional variety. This difference may be associated with variations in root exudate composition among maize varieties, as root exudates or residues from transgenic maize may promote nutrient release during decomposition [20]. Previous studies have reported that Bt maize roots can release higher amounts of organic acids and soluble carbon sources, which may enhance soil enzyme activities and stimulate microbial metabolism, thereby influencing OM accumulation and microbial turnover [21,22,23].

Soil microorganisms are key contributors to the maintenance of soil ecosystem functions and nutrient cycling, and changes in their diversity and community structure reflect the complex and dynamic interactions among plants, soil, and microbes [24]. The expression of exogenous proteins in Bt plants may alter the composition of root-associated metabolites, thereby modifying microbial recruitment and the rhizosphere environment, ultimately influencing microbial community structure [25]. In terms of bulk soil microbial communities, after five consecutive growing seasons, Bt maize cultivation did not significantly affect the α-diversity of bacteria and fungi, and the dominant phyla remained consistent, indicating that Bt maize did not alter the overall diversity of bulk soil microbial communities. Some differences in community composition were observed among Bt varieties; for instance, the bacterial community structure in the 5422CBCL treatment exhibited a slight deviation in principal coordinate analysis, which may be associated with differences in the type and quantity of root exudates among maize varieties, providing distinct nutrient sources and ecological niches that promote the proliferation of specific microbial groups [2]. Soil environmental factors influenced both bacterial and fungal community structures. Among these factors, TP, OM, and TN played key roles in shaping bacterial communities, whereas AN and OM were the major drivers of fungal community structure. Correlation heatmap analysis further revealed significant associations between multiple microbial phyla and these physicochemical factors, highlighting the important regulatory role of the soil environment in microbial community assembly. Overall, Bt maize varieties may indirectly shape microbial community structure by altering the nutrient status of bulk soil, thereby potentially affecting the stability and functioning of the soil ecosystem.

Compared with the bulk soil, significant differences were observed in the Shannon and Simpson indices of the rhizosphere soil bacterial communities among the near-isogenic conventional maize (5422) and the two Bt maize varieties (5422Bt1 and 5422CBCL) after five consecutive growing seasons, whereas the Chao1 and ACE indices showed no significant differences. This indicates that species richness was not reduced, but decreased community evenness led to a decline in overall diversity, thereby driving a restructuring of the rhizosphere soil microbial community [26,27]. Notably, observations over five consecutive growing seasons indicate that the effects of Bt gene insertion on soil bacterial communities are mainly reflected in the modulation of the relative abundances of existing taxa rather than in fundamental alterations in community structure [28]. In contrast, no significant differences were detected in α-diversity indices or community composition of fungal communities among treatments, suggesting a relatively weak response of rhizosphere soil fungi to Bt maize cultivation and a certain degree of structural stability. This stability may be attributed to the inherently broad ecological niches and life-history robustness of fungal taxa [29]. Given the greater complexity of the rhizosphere environment, shifts in microbial communities are likely driven by multiple interacting factors, and the mechanisms underlying the responses of different maize varieties warrant further investigation [30].

In summary, under the conditions of this study, five consecutive seasons of Bt maize cultivation did not exert significant effects on the bulk soil ecosystem, indicating a relatively low ecological risk. Although the rhizosphere soil bacterial community exhibited a certain degree of sensitivity to Bt maize at the diversity level, no fundamental shifts in community structure were detected. Indeed, accumulating field evidence in recent years has demonstrated that Bt maize has limited impacts on soil physicochemical properties, key enzyme activities, microbial diversity, and functional redundancy under multi-season continuous cultivation, providing empirical support for its ecological safety [31,32].

Previous studies have indicated that, in agricultural systems with continuous Bt crop cultivation, certain functional microbial groups—such as phosphate-solubilizing bacteria, nitrifying bacteria, or arbuscular mycorrhizal fungi (AMF)—may undergo shifts in abundance due to selective pressures, potentially affecting soil nutrient cycling and pathogen suppression [33]. In addition, although Bt proteins can be degraded by soil microorganisms, their accumulation dynamics are influenced by soil type, climatic conditions, and management practices. Low but persistent residues of Cry proteins have been detected in some Bt cotton and Bt maize fields [34,35]. Therefore, evaluations of the ecological safety of Bt crops should not rely solely on short-term microbial diversity indicators but should also consider long-term trends in key functional microbial groups, the stability of microbial co-occurrence networks, and soil enzymatic activities as indicators of ecosystem functioning. It should be noted that this study primarily focused on the integrated ecological effects of Bt maize on soil nutrient status and microbial community structure under continuous cultivation, and did not directly measure the residual levels or environmental behavior of Bt toxins in the soil. Future research should build upon long-term field trials to systematically assess the potential impacts of Bt crops on soil ecosystems by combining analyses of Bt toxin residues and degradation dynamics with investigations of functional microorganisms and relevant ecological function indicators, thereby further improving the scientific evaluation framework for the ecological safety of genetically modified crops.

## 4. Materials and Methods

### 4.1. Experimental Materials

The maize varieties used in this study were the transgenic Bt maize 5422Bt1 (Bt11) and 5422CBCL (Mon810) from Beck’s Hybrids, USA, along with their near-isogenic conventional maize 5422. All three varieties were kindly provided by Dr. Cindy Nakatsu from the Department of Agronomy, Purdue University.

### 4.2. Experimental Design

The experiment was conducted in the greenhouse plots of the Department of Ecology, College of Agriculture, South China Agricultural University. The soil physicochemical properties were as follows: pH 6.0, organic matter 34.85 g·kg^−1^, total nitrogen 1.16 g·kg^−1^, total phosphorus 1.28 g·kg^−1^, total potassium 19.42 g·kg^−1^, available nitrogen 95.16 mg·kg^−1^, available phosphorus 68.86 mg·kg^−1^, and available potassium 196.37 mg·kg^−1^. A randomized block design was used for the experiment, consisting of three blocks, each divided into three plots. The three maize varieties (5422, 5422Bt1, and 5422CBCL) were planted separately in each block. Each plot contained eight maize plants, with a plant spacing of 35 cm and a row spacing of 1 m. Sowing was carried out in March (Spring) and October (Autumn) each year, and maize was continuously cultivated for five consecutive seasons. During planting, a basal fertilizer was applied by hole placement at a fixed rate. Specifically, 10 g of Norwegian compound fertilizer (with total nutrient content ≥ 45%, total nitrogen ≥ 15%, available phosphorus ≥ 15%, and available potassium ≥ 15%) was applied between every two maize plants. Additionally, 10 g of the same fertilizer was applied in holes opened about 15 cm outside the outermost maize plants at both ends of each row. During the maize growth period, two additional fertilizations were performed—at the jointing stage and the large trumpet period—using the same application rate as the first. Watering was conducted quantitatively according to the maize growth requirements. After five consecutive planting seasons, soil samples were collected for analysis.

### 4.3. Sampling Method

After five consecutive planting seasons, both bulk soil and rhizosphere soil were collected. For each plot, three maize plants were randomly selected and carefully uprooted with the roots intact. The loosened soil that naturally detached from the roots during gentle shaking was collected in sterile plastic bags and designated as bulk soil. A portion of this soil was stored at −40 °C for microbial high-throughput sequencing, while the remaining portion was air-dried for the determination of soil nutrient contents.

For the collection of rhizosphere soil, sterile gloves were worn to gently remove loosely adhering soil particles from the root surface. The treated roots were then placed into sterile plastic bottles containing an appropriate amount of 0.85% NaCl solution and shaken on a rotary shaker for 20 min to detach the rhizosphere soil. The resulting suspension was centrifuged at 4000 rpm for 5 min, and the supernatant was discarded. Then, 1 mL of 0.85% NaCl solution was added to the pellet, which was resuspended thoroughly using a sterile pipette tip and transferred into a 2 mL sterile centrifuge tube. The sample was centrifuged again at 14,000 rpm for 2 min, after which the supernatant was discarded. The resulting pellet was stored at −40 °C for subsequent high-throughput sequencing analysis of rhizosphere soil microbial communities.

### 4.4. Experimental Methods

#### 4.4.1. Determination of Soil Nutrient Contents

The contents of organic matter (OM), alkaline hydrolyzable nitrogen (AN), available phosphorus (AP), available potassium (AK), total nitrogen (TN), total phosphorus (TP), and total potassium (TK) in the bulk soil were determined following the methods described by Bao [36]. Specifically, OM matter was determined using the potassium dichromate volumetric method; AN was measured by the alkali diffusion method; AP was determined by the sodium bicarbonate extraction method; and AK was measured using the ammonium acetate extraction–flame photometry method. TN was determined by the potassium dichromate–sulfuric acid digestion method, TP by the sulfuric acid–perchloric acid digestion method, and TK by the sodium hydroxide fusion–flame photometry method.

#### 4.4.2. High-Throughput Sequencing of Soil Microorganisms

The total DNA extraction, PCR amplification, MiSeq library construction, and MiSeq sequencing of soil samples were performed by Guangdong Magigene Technology Co., Ltd. (Guangzhou, China). The bacterial 16S rRNA gene (V4 region) was amplified using the primer pair 515F (5′-GTGCCAGCMGCCGCGGTAA-3′) and 806R (5′-GGACTACHVGGGTWTCTAAT-3′) [37]. For fungal community analysis, the internal transcribed spacer (ITS) region was amplified with primers ITS1F (5′-CTTGGTCATTTAGAGGAAGTAA-3′) and ITS2 (5′-GCTGCGTTCTTCATCGATGC-3′) [38].

The PCR amplification was performed in a 20 μL reaction system containing 4 μL of 5× FastPfu buffer, 2 μL of dNTPs (2.5 mM), 1.6 μL each of forward and reverse primers (5 μM), 0.4 μL of FastPfu DNA polymerase, 0.2 μL of bovine serum albumin (BSA), and 10 ng of template DNA. The PCR amplification program was as follows: initial denaturation at 95 °C for 3 min, followed by 30 cycles of denaturation at 95 °C for 30 s, annealing at 55 °C for 30 s, and extension at 72 °C for 45 s, with a final extension at 72 °C for 10 min. The raw sequencing data were processed using QIIME (https://qiime.org/scripts/assign_taxonomy.html, accessed on 25 March 2024) for sequence assembly and quality control filtering. Subsequently, the high-quality sequences from all samples were normalized and analyzed using the USEARCH program, and operational taxonomic units (OTUs) were clustered at a 97% sequence similarity threshold.

### 4.5. Data Analysis

The raw data were organized using Microsoft Office Excel 2019. One-way analysis of variance (ANOVA) was performed with IBM SPSS Statistics 26 to compare differences in soil chemical properties, microbial α-diversity indices, and the relative abundances of major microbial taxa among the three treatments. Microbial α-diversity was evaluated based on the observed number of OTUs, the Shannon index, and the Chao1 index.

After standardizing the soil microbial community data, Bray–Curtis distances were calculated to assess dissimilarities among samples. Principal Coordinates Analysis (PCoA) and Analysis of Similarities (ANOSIM) were performed to evaluate the β-diversity differences in microbial communities among different treatments. Furthermore, Redundancy Analysis (RDA) was conducted to explore the relationships between bacterial and fungal community structures and environmental factors. All figures were generated using Origin 2018 software.

## 5. Conclusions

The results of soil physicochemical analyses showed that Bt maize cultivation did not exert significant effects on nutrient parameters in the bulk soil. Only the OM content in the 5422CBCL treatment was higher than that in 5422Bt1, suggesting potential differences in root metabolic activity between Bt varieties; however, these variations did not alter the overall soil nutrient status. Analysis of the bulk soil microbial communities indicated that the α-diversity of both bacterial and fungal communities did not differ significantly among maize varieties, and the community composition at the phylum level remained highly consistent, demonstrating that continuous cultivation of Bt maize did not modify the microbial community structure in this soil compartment. At the β-diversity level, the overall structures of both bacterial and fungal communities were likewise unaffected. In the rhizosphere soil, Bt maize cultivation resulted in decreased bacterial community evenness and overall diversity compared with the near-isogenic conventional maize 5422, while species richness remained unchanged. In contrast, the diversity and composition of the fungal community showed no significant differences among treatments, indicating a relatively high degree of stability. Both PCoA and correlation analyses further demonstrated that, under continuous cultivation, maize variety had no significant effect on the β-diversity of microbial communities in either the root-surrounding or rhizosphere soil. Overall, continuous cultivation of Bt maize exerted only minor effects on the soil ecosystem. The root-surrounding environment exhibited strong ecological stability, whereas the observed changes in the rhizosphere soil bacterial community highlight the need to pay closer attention to compartment-specific microbial responses in ecological safety assessments. This study provides evidence supporting the ecological safety of Bt maize under continuous cultivation and emphasizes the importance of integrating continuous cultivation field monitoring with functional microbial analyses to further refine ecological risk assessment frameworks for genetically modified crops.

## Figures and Tables

**Table 1:** Nutrient content of bulk soil.

Sample	OM (g/kg)	TN (g/kg)	TP (g/kg)	TK (g/kg)	AN (mg/kg)	AP (mg/kg)	AK (mg/kg)
422	26.12 ± 0.53 ab	1.25 ± 0.04 a	1.80 ± 0.06 a	21.34 ± 0.30 a	73.88 ± 6.86 a	241.14 ± 13.72 a	566.96 ± 40.50 a
5422Bt1	24.01 ± 0.74 b	1.26 ± 0.05 a	1.81 ± 0.04 a	21.08 ± 0.40 a	75.21 ± 2.63 a	239.36 ± 6.65 a	598.11 ± 59.60 a
5422CBCL	28.11 ± 1.46 a	1.21 ± 0.05 a	1.80 ± 0.07 a	21.28 ± 0.24 a	89.46 ± 3.74 a	228.10 ± 3.62 a	658.15 ± 58.04 a

Note: OM—organic matter; TN—total nitrogen; TP—total phosphorus; TK—total potassium; AN—alkaline hydrolyzable nitrogen; AP—available phosphorus; AK—available potassium. Different letters (a and b) indicate significant differences among treatments according to one-way ANOVA (Duncan’s test, *p* < 0.05).

## Data Availability

The original contributions presented in this study are included in the article. Further inquiries can be directed to the corresponding author.
